# A Lightweight Data Integrity Scheme for Sensor Networks

**DOI:** 10.3390/s110404118

**Published:** 2011-04-07

**Authors:** Ibrahim Kamel, Hussam Juma

**Affiliations:** 1 Department of Electrical and Computer Engineering, University of Sharjah, P.O. Box 2727, Sharjah, UAE; 2 Dubai World, P.O. Box 4399, Dubai, UAE; E-Mail: hussam.mohammed@dubaiworld.ae

**Keywords:** wireless sensor network, security, data integrity, watermarking

## Abstract

Limited energy is the most critical constraint that limits the capabilities of wireless sensor networks (WSNs). Most sensors operate on batteries with limited power. Battery recharging or replacement may be impossible. Security mechanisms that are based on public key cryptographic algorithms such as RSA and digital signatures are prohibitively expensive in terms of energy consumption and storage requirements, and thus unsuitable for WSN applications. This paper proposes a new fragile watermarking technique to detect unauthorized alterations in WSN data streams. We propose the FWC-D scheme, which uses group delimiters to keep the sender and receivers synchronized and help them to avoid ambiguity in the event of data insertion or deletion. The watermark, which is computed using a hash function, is stored in the previous group in a linked-list fashion to ensure data freshness and mitigate replay attacks, FWC-D generates a serial number *SN* that is attached to each group to help the receiver determines how many group insertions or deletions occurred. Detailed security analysis that compares the proposed FWC-D scheme with SGW, one of the latest integrity schemes for WSNs, shows that FWC-D is more robust than SGW. Simulation results further show that the proposed scheme is much faster than SGW.

## 1. Introduction

A WSN typically consists of base stations and a number of wireless sensors. Sensors are usually small in size, have limited computing capabilities, communicate wirelessly and are powered by small batteries. These sensors are often scattered in a sensor field. Data from the sensor field is collected and sent to a base station. The base station then sends the data to the end users for analysis and strategic decisions. Base stations usually have unlimited power, sufficient memory, powerful processors and a high bandwidth link, in comparison to other sensor nodes [1].

WSNs are used in many fields. For example, WSNs are used in military applications for monitoring friendly forces, battlefield surveillance, biological attack detection, troop coordination, and battle damage assessments. In environmental applications, sensors can be used to detect and monitor environmental changes like tracking oil pollution.

Data integrity is a core requirement for secure sensor data in WSN. False or malicious data would result in incorrect decisions and potentially financial losses. One of the major security challenges for WSNs is the conflict between the limited resources, e.g., computational capabilities, available power, and storage capacity at one hand and security requirements at the other hand. Most of the prior works on securing sensor networks use traditional security solutions that are based on cryptographic algorithms [1–3]. These techniques usually execute thousands or even millions of multiplication instructions in order to perform operations like modular exponentiation. Consequently, they are too expensive and not suitable for sensors [4,5].

A digital watermark is a lightweight technique that was used traditionally for providing copyright protection for multimedia data like images and video clips. Watermarking algorithms are much lighter and thus require less battery power and processing capabilities than cryptographic-based algorithms. Another advantage for the watermarking-based algorithms is that the watermark is embedded directly into the sensor data; there is no increase in the payload. While cryptography provides no protection after the content is decrypted, watermarking provides protection in secrecy at all times because the watermark is an inseparable constituent part of the host media [6–8]. The main idea of digital watermarking is to embed a piece of secret information (the watermark) into the data stream in such a way that any change or tampering with the original data would corrupt the watermark. This type of watermarking is called *fragile watermarking*, as opposed to the *robust watermarking,* which is used mainly for copyright protection [9–11].

This paper proposes a lightweight simple watermarking scheme to protect sensor data against possible malicious attacks. The main contributions of this paper are as follows:

- Propose a lightweight fragile watermarking scheme (FWC-D) to provide data integrity for WSNs.
- Provide detailed security analysis for the proposed technique and compare it with the SGW [12].
- Perform simulation experiments to measure the execution time of the proposed techniques and compare it with the SGW data integrity scheme.

The remainder of this paper is structured as follows. Section 2 discusses the threat and attack model considered in this paper. Section 3 summarizes some of the related works in WSN security. The proposed data integrity scheme is presented in Section 4. A detailed security analysis for the proposed technique is presented in Section 5. Section 6 provides simulation experiments that measure the execution cost of the proposed scheme and compares it with the execution cost of SGW data integrity scheme. Conclusions and future works are presented in Section 7.

## 2. Threats and Attacks Model

The nature of wireless communication makes wireless networks more vulnerable to attacks than wired networks. Moreover, WSNs are often deployed in uncontrolled environments, which make it susceptible to physical tampering. The limited computational capabilities and energy resources are additional challenges that need to be dealt with in designing security scheme for WSNs. The following are the main threats on the integrity of the WSN data [13–15]:

*Data Modification Attack*: An adversary modifies the value of one or more the data readings either by hijacking the sender sensor or inserting itself between the sender and receivers.

*False Data Insertion*: An adversary can compromise existing nodes and inject a false message with false information. It is also possible that the adversary add new nodes to the sensor networks that feed false data. Such attack also consumes the energy resources of other sensor nodes.

*Data deletion*: Data deletion attack can take place by dropping individual data readings or dropping one or more groups and preventing them from reaching to the intended recipient.

*Denial of Service*: Denial of service attacks on a wireless sensor network may take on several forms, e.g., disrupting the radio link, misroute sensor data, or exhaust node resources. Section 5 shows how the proposed technique deals with each of the above attacks.

## 3. Literature Review

In this section we present a summary of the previous work that is related to data integrity in wireless sensor networks. Effort for securing WSNs have mainly focused on providing confidentiality and integrity services [16,17]. Confidentiality services protect critical data from eavesdropping by unauthorized users. Confidentiality is usually achieved by encrypting the data using a secret key. While an important part of overall security, data confidentiality is outside of the scope of this paper. In this paper we focus on the problem of providing data integrity in sensor networks. Data integrity services ensure that the received data is in the form intended by the originator and has not been altered in transit. Most of the prior works on securing sensor data use traditional security solutions that are based on cryptographic algorithms such as SPINS [18], TinySec [19] or LEAP [20]. Cryptographic-based algorithms usually employ expensive operations like exponentiation and modular arithmetic of large numbers. However, because of the inherent resource limitations in wireless sensor nodes cryptographic-based algorithms are not suitable for WSNs applications. In this paper we use a watermarking-based technique for protecting the integrity of WSNs. The advantage of watermarking-based algorithms is that they are much lighter and require less power and processing capabilities than cryptographic-based algorithms.

Digital watermarking techniques can be classified into *fragile* and *robust*. In *robust watermarking* schemes the watermark is designed such that it can stand malicious attacks like deletion, modification, and cropping. *Robust watermarking* is used mainly for copyright protection [21,22]. Fragile watermarking embeds the watermark (secret message) into the data stream in such a way that any change or tampering with the original data would corrupt the watermark. This paper proposes fragile watermarking scheme to ensure data integrity in WSNs.

Perrig *et al.* [2,18] introduced the SPINS algorithm, which consists of two security protocols: the Sensor Network Encryption Protocol (SNEP) and Micro Timed Efficient Stream Loss-tolerant Authentication (μTESLA). The function of SNEP provides confidentiality (privacy), two-party data authentication. μTESLA, which is an adoption of the TESLA protocol [23] provides integrity and freshness. μTESLA attaches a MAC to each packet transmitted. One of the important ideas here is that the key used to create the MAC is not sent with the packet. In μTESLA the base station generates a sequence of secret keys (one-way key chain) of length *n* right-to-left by repeatedly using a public cryptographic one-way function *F*. Each authentication key is part of a key chain. Each key of the key chain is associated with a time interval and all packets sent within one time interval are authenticated with the same authentication key. When a sensor node receives a packet, it cannot verify the MAC as the authentication key has not yet received. The receiver node stores the packets in a buffer until the authentication key is disclosed (based on the time schedule for disclosing keys). When the key is disclosed, the receiver can verify the correctness of the disclosed key. If the disclosed key is authentic, the node can use it to authenticate the packet stored in its buffer. Otherwise, the receiver needs to drop the unsafe packet because an attacker might have altered it during the transmission. Unlike our proposed method, μTESLA cannot figure out the number of packets that are inserted or deleted by the attacker. μTESLA is also vulnerable to DoS attacks; an attacker may jam key disclosure packets to saturate storages of sensor nodes. From the processing cost point of view, key setup is considered expensive and requires non-trivial memory for storing the key chain. μTESLA also requires tight time synchronization which is expensive for wireless sensor networks.

Albath and Madria [24] proposed a security scheme for data streams called PADS to provide integrity and protection against passive eavesdropping by applying confidential transmissions of data messages. In their scheme, a one-time pad (OTP) is computed by the sensor node using the sensor reading, with the secret key being shared between the sensor, base station, and MAC. The Message Authentication Code (MAC) is calculated and attached to the data packet in order to achieve data integrity. Then the OTP is XORed to the data reading in order to provide protection against eavesdropping. One problem with PADS is that like μTESLA it cannot determine how many packets are inserted or deleted. PADS also assumes that the base station and the sensors are time synchronized, which is too expensive for wireless sensor networks and makes it vulnerable to delay attacks. In the delay attack, a malicious attacker can hold a data packet for a certain period and release it later to the receiver. Delay attacks might be combined with inserting one or more data packets and in such a case the receiver cannot determine whether the mismatch occurs because of insertions or because of modification attacks.

TinySec [19] is a linked layer security protocol that provides data confidentiality, data integrity and data authentication. To guarantee message integrity and authenticity, TinySec computes a four-byte message authentication code (MAC) over the packet. The MAC would detect any tampering for the transmitted data. Zhu *et al.* [20] proposed LEAP, a key management scheme for sensor networks to support and restrict the security impact of nodes in the immediate neighborhood of the compromised node. LEAP uses four types of symmetric keys for each sensor node that can be used for providing authentication and confidentiality in WSNs. The four keys are: an individual key that is shared with the base station, a pair-wise key shared with another sensor node and used to send messages with the required privacy, a cluster key shared with multiple neighboring nodes for securing local broadcasts, and a group key that is shared among all the sensor nodes in the network and is used to send broadcast messages. The main problem with LEAP is its high processing and communication cost. It also requires nontrivial memory for storing all keys, especially when the size of the WSN increases.

Sion *et al.* [22] have described a robust watermarking scheme for streaming data proposed for copyright protection. Streams are defined as a continuous sequence of numerical values. The technique identifies key points in the stream called major extremes. A set of major extremes in the group, are identified and selected such that these extremes will survive uniform sampling. The watermark bits are embedded in the major extremes. Thus, trying to destroy the watermark would leave the stream not useful. The watermark can be later extracted and used to proof copyright and ownership of the data stream.

Guo *et al.* [12] proposed a security scheme to provide integrity for sensor data, which is referred to as Sliding Group Watermark (SGW) in this paper. In SGW, the data streams are split into groups of variable size. The size of the group is determined adaptively as a function of the data itself. A secure hash function is applied for each data element in the stream and if the hash value is zero, then the data element is called a synchronization point (marks the end of the group). SGW scheme computes the secure hash function several times. First, the secure hash function is calculated at the data element level, then at the group level. Finally, the secure hash is computed for every two consecutive groups. Another problem with the SGW is that the insertion, modification, and deletion attacks may create confusion at the receiver side. When such attacks occur the receiver may lose track of the synchronization points. By forming the wrong groups at the receiver side, watermark checks will fails, which results in rejecting authentic data readings. In [25] we proposed LWC (light-weight chained watermarking) scheme, which, simplifies the SGW and avoids several of its drawbacks. LWC uses chained watermarks; however, it is less complex than SGW. LWC provides significant performance improvement (one to two orders of magnitude in computational overhead over the SGW technique). However, LWC suffers from the same security holes that SGW has. The proposed scheme avoids the security weaknesses in SGW and LWC and at the same time enjoys less computation overhead. Section 5.1 presents detailed security analysis that highlight the superiority of the proposed FWC-D over the SGW and LWC techniques.

Other watermarking techniques have been developed for relational databases, e.g., [1] and [21] for copyright protection of databases and [27,28] for data integrity protection. However, these schemes require that the entire database table to be available at the time of watermark embedding, thus, they are not suitable for streaming data [29].

## 4. The Proposed Solution FWC-D

The goal is to develop an efficient data integrity scheme, which is fast and lightweight. FWC-D embeds a piece of secret information (the watermark) into the data stream in such a way that any change or tampering with the original data corrupts the watermark. The proposed scheme organizes the sensor data readings into groups of constant sizes.

The proposed scheme uses the hash function *HASH()*, which is applied to the concatenation of all individual data elements in the group along with the secret key *K* to compute the watermark. *HASH()* can be any secure hash function such as MD5 or SHA. In our implementation, we use the MD5 (Message Digest 5) algorithm, which produces a fixed size number—128 bits (32 hex numbers). The watermark is embedded in the least significant bits of the data items (in our implementation the last bit of each data reading is ignored when computing the hash value). Thus, applications are expected to tolerate small distortions [1]. The watermark is stored in the previous group to make it more difficult for the attacker to insert or delete a complete group without detection. Since the attacker does not know the secret key K any modifications made to the data can be detected.

Using the secret key, the receiver can extract the watermark (calculated at the sender side) from the received data. To verify the integrity of the received group, the receiver recalculates the watermark and checks against the extracted watermark. If the two watermarks match, the group is considered authentic; in case of a mismatch, the group is reported as not authentic. The proposed scheme consists of three main processes:

- Organizing data readings into groups with constant sizes.
- Watermark generation and embedding algorithm at the sender side.
- Watermark extraction and integrity check algorithm, to check and verify the integrity of the received groups.

In general different sensors are working independently and are not synchronized. FWC-D uses group delimiters to keep the sender and receiver synchronized (in recognizing the beginning and the end of the group) and avoids any ambiguity in case of data insertion or data deletion. The delimiter value *D* can be any value that does not occur in the data readings. For example, if the sensors measure atmosphere temperature, the delimiter value *D* can be FF hex (since atmosphere temperature cannot reach 128 °C). FWC-D uses constant group sizes. Notice that using variable group size is not useful as the malicious observer can figure out the group size easily by following the delimiter. Thus the proposed security scheme organizes data readings into groups of fixed size.

FWC-D generates a serial number *SN* that will be attached and stored with each group. This will help the receiver to determine how many group insertions or deletions have occured in case of group insertion or deletion attacks. The watermark of group *g_i_* in FWC-D is formed by applying a secure hash function *HASH()*. Secure hash function adds a secret key *K* to the concatenation of the data elements in group *g_i_*. Thus the watermark *W* is formed as follows:

(1) $$W_{i}=\mathrm{HASH}\left(K\left\|g_{i}\right\|\mathrm{SN}\right)$$

To prevent the SN from increasing indefinitely, we limit its size to *x* bits. Consequently groups will take numbers from 0 to *2^x^ − 1*. After SN reaches to *2^x^ − 1*, it is reset back to 0. A set of data groups that are numbered from 0 to *2^x^ − 1* are referred to as a *segment*.

The overhead of the serial number and group delimiter value is expected to be negligible when compared to the size of the group. For example, if the group size = 50 readings and 10 bits are used for the serial number *SN*, then the overhead is 0.2 bit/data element. Note that secure hash functions, like MD5, guarantees that the probability of birthday attacks and collision attacks are very low as long as the message size is less than 2^64^. This means that, theoretically, one can use very large group sizes. Newer secured hash functions like SHA-160, SHA-256 and SHA-512, which allow up to 2^128^.

**Figure 1.**
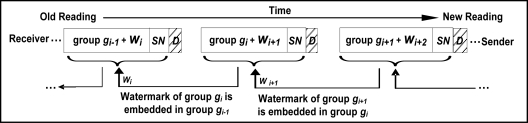
*Forward-chaining* Watermark Embedding Process (Default).

**Figure 2.**
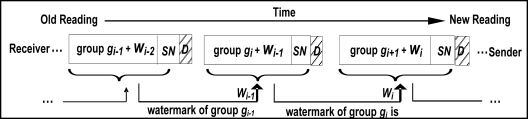
Backward-chaining watermark embedding process.

To thwart replay attacks and to ensure data freshness, FWC-D does not send the watermark with the corresponding group of data readings, but rather it embeds the watermark in the earlier group. Thus, the watermark *W_i_* of group *g_i_* is sent with group *g_i−1_* as shown in Figure 1. This way, the watermark is chained across all groups, making it more difficult for an attacker to copy one or more data groups and replay them later. This can be achieved at the expense of some additional buffer requirements at the sender and receiver sensors. Storing watermarks in the earlier group (*W_i_* of group *g_i_* is sent with group *g_i−1_*) is referred to as *forward-chaining*. Alternatively, the watermark can be stored in the following group (*W_i_* of group *g_i_* is sent with group *g_i+1_*) as shown in Figure 2. This setup is referred to as *backward-chaining*.

From the security analysis point of view, *forward-chaining* and *backward-chaining* have the same effect on the robustness of the security scheme. The difference is in the buffer overhead at the sender and the receiver. In *forward-chaining*, the sender needs two buffers to store the current group and the previous group. At the receiver end only one buffer is needed. On the other hand, with *backward-chaining*, where the watermark of the current group is stored in the following group the sender needs only one buffer, while at the receiver side two buffers are needed.

One of the popular application scenarios is having small sensors that operate on battery power sending data readings to a receiver (the base station), which is a relatively powerful machine that is connected to a continuous power source (e.g., the wall power outlet). In this case, *backward-chaining* is preferable over the *forward-chaining* since the base station is more powerful and can afford to have a large buffer size.

The group size *Z* is a design parameter and it depends on the capability of the sensors used. There is a trade off in selecting the group size *Z*. The use of small group increases the distortion introduced to the each data reading and the payload overhead. On the other hand, a large group size requires a larger buffer to store the data elements of the group and it increases the watermark computation time.

The group size is determined by the application and the power of available sensors. Some applications use high end sensors that are connected to permanent power supplies (e.g., work on wall power) and equipped with powerful CPUs and large amounts of memory. In such cases a large group size is useful as it minimizes the overhead and increases the probability of attack detection. On the other hand, in the case of small and weak sensors where computing and power capabilities are very limited, a smaller group size is desirable. Furthermore, if the application requires a high rate of transmission then the latency should be small and consequently a small group size should used.

### 4.1. FWC-D Embedding Algorithm

The sender keeps buffering data readings until one complete group, say *g_i_* of size Z, is formed. The watermark *W_i_* of group *g_i_* is computed using the secure hash function *HASH()*, which is applied to the concatenation of all individual data readings in the group along with secret key *K* and group serial number *SN*. In the following discussions we assume a *forward-chaining* watermarking scheme in which the watermark is stored in the earlier group of data. Thus, the sender has to buffer two groups of data readings, say *g_i_* and *g_i+1_*. The sender computes *W_i+1_* the watermark of *g_i+1_* and embeds it in *g_i_* by replacing the least significant bits in the data readings. Once the watermark is embedded, group *g_i_* is sent along with the serial number *SN* and group delimiter *D* to the receiver.

Like other watermarking techniques that are based on least significant bit replacement, the underlying application should be able to tolerate small distortions introduced to the data readings. By replacing the least significant bits, the values of data reading might change by one. If two bits are replaced, the value of the data readings might change by up to three. The size of the watermark (in terms of the number of bit) is constant and it depends on the secure hashing function. In the case of MD5 it is 128 bits. Thus if the group size *Z* = 128, then each bit from *W_i_* can be placed in one data reading in the group. If the size of *W_i_* is smaller than the group size *Z*, then at most one bit per data reading is replaced. In this case, the data readings that will carry the bits of *W_i_* can be selected carefully (in agreement with the receiver) to make it harder on the attacker to figure out the watermark. However, if *W_i_* is larger than *Z* then one possibility is to store more than one bit of *W_i_* per data reading. However, the distortion introduced to the data reading increases with increasing number of bits replaced. Note that using part of the hash value as a watermark might compromise the security guarantee of the secure hash function. In this case it is better to use larger group size to provide the required security guarantees.

### 4.2. FWC-D Detection Algorithm

The receiver keeps buffering data readings as they arrive until the group delimiter is encountered. To make sure that the data are integral and have not been altered, the receiver reconstructs the watermark by calculating the hash value of each group it receives and compares it with the watermark (hash calculated at the sender side) sent by the receiver. If the two hash values match, the data readings are considered integral and they are accepted by the receiver.

**Figure 3.**
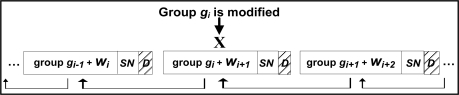
Data Modification Attack in *Forward-chaining* Watermarking Scheme.

Remember that because we are assuming *forward-chaining*, the watermark *W_i+1_* of group *g_i+1_* is sent with the group *g_i_* as shown in Figure 3. Thus when the sender receives group *g_i_*, it extracts *W_i+1_* and buffers it until group *g_i+1_* arrives. The receiver sensor should have enough storage to buffer one data group and the watermark of the following group. Upon the arrival of all data readings that belong to group *g_i+1_*, the watermark of group *g_i+1_* is then reconstructed by applying *HASH()* to the concatenation of all individual data readings in the group *g_i+1_* along with secret key *K* and group serial number *SN*. Then the reconstructed watermark is checked against the extracted watermark from group *g_i_*. If the two watermarks match, the data readings of *g_i+1_* are accepted and otherwise they are rejected.

In addition, the receiver checks the serial number of the received group to check for deleted or inserted groups. Section 5 discusses in more detail how the receiver can detect various types of attacks.

## 5. Security Analysis of the FWC-D Scheme

An attack is considered successful if it is not detected by the receiver. In this section we discuss various types of attacks that can be lunched in the wireless sensor network scenario and how the proposed security scheme can be used to thwart these attacks.

**Data Modification**: Let us assume that the attacker has altered the content of group *g_i_*. Note that alteration might affect one or more of the group *g_i_* constituents, e.g., the value of one or more of the data elements of the group, the serial number, the delimiter, or even the watermark of group *g_i−1_*. Next we will briefly discuss each of these scenarios.

The first scenario is the case where modification affects the least significant bits of group *g_i_*. Recall that the least significant bits of group *g_i_* contain the *W_i−1_* (watermark of the *g_i+1_* group). At the receiver end, the integrity check of group *g_i+1_* will fail, even though the attacker has not altered group *g_i+1_*, and thus, group *g_i+1_* will be rejected by the receiver. On the other hand, the integrity check for group *g_i_* succeeds because the calculated hash value matches the extracted watermark *W_i_* that was shipped with group *g_i−1_* (note that the least significant bit of the data readings is not included in the watermark calculation). As a result, the receiver will accept group *g_i_*.

In the second scenario, we assume that the attack altered the value of one or more data readings from group *g_i_*. Further we assume that the attack does not change the least significant bits. As a result, the receiver will reject group *g_i_* because the calculated watermark does not match the one sent by the receiver. At the same time, since the least significant bits of the group *g_i_* (that contains the watermark of group *g_i+1_*) are integral, thus, the receiver will consider *g_i+1_* authentic and accept it. If the attacker changes both the data and the least significant bits of group *g_i_* (*W_i+1_*) as a result, the integrity check of groups *g_i_* and *g_i+1_* will fail and the receiver will reject both groups.

In the third scenario, the modification attack only affects the group serial number *SN*. Since the group serial number is included in the watermark computation, the integrity check of the victim group will fail. As a result, the receiver will reject group *g_i_*. But since the least significant bits of group *g_i_* have not been affected, so the receiver accepts *g_i+1_*.

In the fourth scenario, the modification affects the group delimiter only. The receiver will continue reading until it encounters the delimiter of the *g_i+1_* group. In this case the receiver will consider groups *g_i_* and *g_i+1_* as one group, but since the group size is greater than the agreed upon group size *Z*, the receiver will reject groups *g_i_* and *g_i+1_*. Moreover, because of the chained watermark, the receiver will not be able to check the integrity of the group *g_i+2_*. The receiver should be able to read and authenticate the group *g_i+3_* and the later groups.

The above attack scenarios are independent and thus if two or more of the above scenarios occur together, the security analysis is this case can be easily derived from the above discussions.

**Data Element Insertion Attack**: Since the group size is constant, the receiver expects to receive exactly *Z* elements before encountering the group delimiter *D*. In case of data element insertion, the receiver will realize that one or more data elements have been inserted. Of course, the receiver will then reject group *g_i_* as there is no way to identify the false data reading. Now, since *W_i+1_* (watermark of group *g_i+1_*) is stored in group *g_i_* the receiver will extract an incorrect *W_i+1_* and as a result rejects *g_i+1_*. The following group, *g_i+2_*, should not be affected as the receiver can extract *W_i+2_* correctly from *g_i+1_*. Group *g_i+2_* will match its watermark *W_i+2_* and it will be accepted. The group *g_i+3_* and later groups will not be affected.

**Data Element Deletion Attack**: In this analysis, we assume that the attacker has deleted one or more data readings from the group *g_i_*. There are two scenarios depending on whether the deleted object was data reading or delimiter *D*:

*Scenario 1:* the deleted data does not include the group delimiter *D*. The receiver can detect that the number of received data elements is less than the group size. As a result, the receiver will reject group *g_i_* and *g_i+1_* will also be rejected because *W_i+1_* is stored in group *g_i_*; the receiver will not be able to check the integrity of group *g_i+1_*. However, since group *g_i+1_* has not been altered, group *g_i+2_* will match its watermark *W_i+2_* and it will be accepted.

*Scenario 2:* we look at the possibility that the deleted data elements include the group delimiter *D*. In this case the receiver will not detect the end of group *g_i_* and consequently join groups *g_i_* and *g_i+1_*. As a result, the receiver will reject both of them. Moreover, since *W_i+2_* (the watermark of group *g_i+2_*) is stored in group *g_i+1_* the receiver will not be able to verify the integrity of group *g_i+2_* and therefore will reject *g_i+2_*. However, since group *g_i+2_* is not altered, then group *g_i+3_* will match its watermark *W_i+3_* (that was sent with group *g_i+2_*) and thus *g_i+3_* and the following groups will be accepted.

In general if the attack affects *f* delimiters where *f* >= 2, *f + 1* groups will be rejected by the receiver.

**Figure 4.**
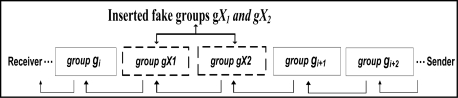
How FWC-D resists Groups Insertion Attack.

**Group Insertion Attack**: In this analysis, we assume that the attacker has managed to insert two groups, *gX_1_* and *gX_2_* (shown in dotted lines in Figure 4) between group *g_i_* and *g_i+1_*. Moreover, let us also assume that the inserted groups have the correct group size and group delimiter. At the receiver end, the integrity of group *g_i_* is already checked. Since the attacker does not have the secret key *K*, which is known only to the sender and receivers, the reconstructed watermark of group *gX_1_* will not match the extracted watermark from group *g_i_*. Note that the receiver will follow the usual procedure and extract the watermark for *gX_1_* from the least significant bits of *g_i_*. Moreover, the reconstructed watermark of group *gX_2_* will not match the extracted watermark from group *gX_1_*. The attacker can generate a hash value for *gX_2_* but because the attacker does not know the secret key *K*, he cannot generate the correct watermark. The insertion of the groups, *gX_1_* and *gX_2_*, will affect the integrity verification of group *g_i+1_*. Thus the receiver will reject group *g_i+1_*. When group *g_i+2_* is formed, the reconstructed watermark of group *g_i+2_* will match the extracted watermark from group *g_i+1_* and thus the receiver will accept group *g_i+2_* as well as later groups of data. The successful integrity verification of group *g_i+2_* will confirm that there are at least two groups that have been inserted between group *g_i_* and *g_i+2_* that have been inserted. Remember that the receiver keeps track of the last serial number it receives. As a result, the receiver will reject groups *gX_1_*, *gX_2_* and *g_i+1_* and accept group *g_i+2_* and the later groups.

**Figure 5.**
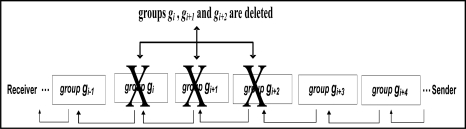
Group Deletion Attack.

**Group Deletion Attack**: To make the description easier we show an example in which three groups have been deleted. The analysis can be generalized to describe the case of deleting and number of groups. Let us assume that the group *g_i_*, *g_i+1_*, and *g_i+2_* have been deleted by the attacker (see Figure 5). Note that the deletion of group *g_i+2_* will also affect the integrity verification of group *g_i+3_* since *W_i+3_* is stored in group *g_i+2_*. At the receiver end, we assume that the integrity of group *g_i−1_* is already verified. Since *g_i_*, *g_i+1_*, and *g_i+2_* have been deleted the receiver gets *g_i+3_* right after *g_i−1_*. Then the receiver goes ahead as usual and computes the watermark of group *g_i+3_* and compares it with the watermark extracted from the previous group *g_i−1_*. The reconstructed watermark of group *g_i+3_* will definitely not match the extracted watermark from group *g_i−1_*. The receiver will consider group *g_i+3_* as unauthentic and reject its data readings. The receiver also extracts the watermark *W_i+4_* from *g_i+3_* and stores it in the memory until the following group arrives (in this case *g_i+4_*). When group *g_i+4_* is formed, the computed watermark of group *g_i+4_* will match the extracted watermark from group *g_i+3_* and thus the receiver will accept group *g_i+4_* as authentic. The successful integrity verification of group *g_i+4_* will confirm that groups *g_i_*, *g_i+1_* and *g_i+2_* have been deleted. As a result, the receiver rejects group *g_i+3_* and accepts group *g_i+4_*.

**Figure 6.**
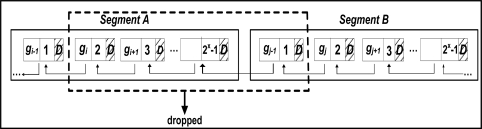
FWC-D: Segment Deletion Attack Scenario.

**Segment Deletion Attack**: Recall the segment refers to a set of successive groups, which have serial numbers, *SN*, from 0 to *2^x^ −1*. Here we assume that the attacker has managed to delete *2^x^ − 1* successive groups as shown in Figure 6. In this case the receiver will see an ordered serial number *SN* in spite of the deletion attack. Let us assume, for example, that the receiver sees group *g_i−1_*, which belongs to segment *A* followed by the group *g_j_*, which belongs to segment *B*. At this point, the receiver detects a mismatch between the reconstructed watermark for group *g_j_* and *W_j_* (the watermark that is extracted from group *g_i−1_*). However, the receiver would not know if the mismatch is caused by a modification attack or a deletion of multiple groups, thus the receiver will reject *g_j_* even though the receiver will not know how many segments has been deleted. However, the receiver recovers from the attack, since the later group *g_j_* is not altered, so group *g_j+1_*, which belongs to segment *B* will match its watermark *W_j+1_* with the one extracted from group *g_j_*. Thus, the receiver will accept group *g_j+1_*, which belong to segment *B* as authentic. The following groups are unaffected by the attack and will be processed as usual.

### 5.1. Comparison with the SGW Watermarking Schemes

The main limitation of SGW [12] is that under some insertion, modification, and deletion attack conditions the receiver loses track of the synchronization points. We recall that in SGW, the synchronization points are used to mark the end of the groups. Therefore, the receiver will not be able to construct the group correctly. Consequently it will compute the wrong watermark and reject data readings. The problem is that this confusion will continue forever. This means that one attack can cause all the following data readings to be rejected by the receiver and it never recovers.

Recall that SGW uses variable group size, which depends on the value of data readings. SGW calculates the hash of each data reading. Data reading is considered a synchronization point if:

1. The modulus of its hash equal to zero and
2. The group has already more than *L* data readings,

where *L* is a predefined threshold. Note that because of the second condition there might be data readings inside the group that fulfill the first condition but they are not considered synchronization points by the sender sensor. If the same data readings arrive at the receiver side (without alteration), the receiver will apply the same two conditions and gets the same set of synchronization points and consequently the same group structure. The problem arises when the group is altered by an insertion, deletion, or even modification attack. The receiver, in this case, may choose synchronization points that are different than those chosen by the sender sensor. This will cause the receiver to form groups that are different than those formed at the sender and the integrity checks fail. The following example will explain this point further.

**Figure 7.**
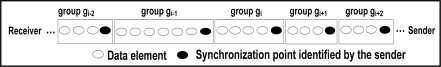
Group formation in SGW watermarking technique.

For example, if the attacker modifies the last data element in group *g_i_*, which is the synchronization point of the group, the modified data elements may become a non-synchronization point. Figure 7 shows five groups of data readings formed according to SGW technique.

**Figure 8.**
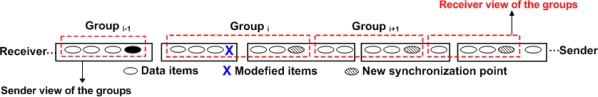
SGW watermarking technique is vulnerable to confusion in forming the groups at the receiver.

The modified data reading is marked by X in the figure. In this case we have two possibilities (in this example we consider the minimum group size *L* = 4):

- If no element from *g_i+1_* satisfies the first condition for the synchronization point, group *g_i_* is combined with group *g_i+1_*. Note that the second condition for the synchronization point will be satisfied after adding at most one new element to *g_i_*.
- If there is at least one element from *g_i+1_* that satisfies the first condition for the synchronization point (shown as a gray oval in Figure 8), the receiver will continue adding data readings (from group *g_i+1_*) to *g_i_* until reaching that element, which will serve as a new synchronization point for *g_i_*.

Moreover, the size of group *g_i+1_* might fall below *L*, so in order to meet the second condition above, the receiver will add data readings (from group *g_i+2_*) to *g_i+1_* to complete at least *L* data readings in group *g_i+1_*. In Figure 8 the dotted line shows the new groups formed at the receiver side (receiver view of the groups), while the solid line shows the sender view of the groups. Unfortunately, this confusion in forming groups at the receiver side might continue forever.

Note that even if the modified data reading is not the synchronization point, the new value of the attacked data element might make it a synchronization point. So the receiver would end the current group incorrectly and group miss-synchronization continues. It can be easily shown that similar scenarios may arise under insertion and deletion attacks but these are not discussed because of the space limitation.

## 6. Performance Evaluation

We have performed experiments to measure the performance and overhead of the proposed watermarking scheme and compare it with the SGW [12]. Experiments were performed using synthetic data streams. Section 6.1 evaluates the performance of the embedding algorithm at the sender sensor while Section 6.2 show the performance of the watermark extraction and integrity check algorithms at the receiver sensor. We used Java JDK 6, Eclipse Platform Version: 3.3.1.1 to implement all algorithms and the simulation environment. All experiments were conducted on an Intel Pentium processor system at 1.86 GHz, with 512 MB of memory and using the Windows XP operating system.

### 6.1. Performance Evaluation of the FWC-D Scheme

**Figure 9.**
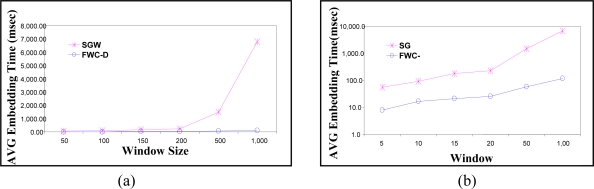
Execution time of the Embedding algorithm at the sender Side. (**a**) Linear Scale, (**b**) Logarithmic Scale.

We calculate the watermark and embed it in one group of data. The average embedding response time is calculated over 30 groups. The first experiment compares the average embedding time of the proposed FWC-D scheme is compared with the SGW scheme [12]. In Figure 9(a) the Y-axis represents the average embedding response time as the data arrives at the sensor side, while the X-axis represents the window size. We changed the group size from 50 to 1,000 readings. The figure shows that on the average SGW is about 59 times slower than FWC-D. The gain in the performance increases with increasing the window size. However, the difference in the performance is not clear in the linear scale when the group sizes are small. Figure 9(b) shows the same experiments on a semi-logarithmic scale to highlight the performance gain when the group sizes are small.

**Figure 10.**
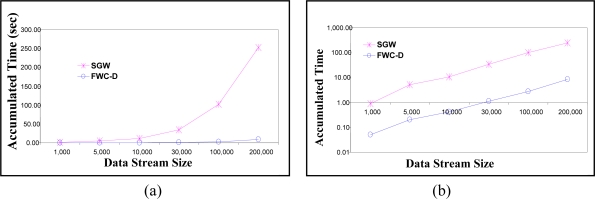
Accumulated execution time of the Embedding algorithm. (**a**) Linear Scale (**b**) Logarithmic Scale.

Figure 10 shows the accumulated execution time of the watermark generation and embedding algorithms for the proposed FWC-D scheme compared to the SGW scheme. The group size *Z* is set to 200. The accumulated execution time of the FWC-D algorithm is the total time used for performing the following operations: constructing the data group, generating and attaching the group delimiter *D* serial number *SN* at the end of each group, watermark computation of embedding the computed watermark.

The importance of the accumulated execution time is that it gives an indication of the expected power consumption at the sensor. The Y-axis represents the accumulated processing time as the data arrives and the X-axis represents that number of data readings sent since the start of the simulation. From the figure, one notices that as the data stream size increases the accumulated execution time of the SGW scheme increases, however, FWC-D scheme at a much lower rate. This means that the power requirement for FWC-D is much less than the power required for executing the SGW scheme and thus, due to resource constraints in WSNs, the FWC-D technique is more suitable for WSNs.

### 6.2. FWC-D Watermark Extraction and Integrity Check Algorithm

**Figure 11.**
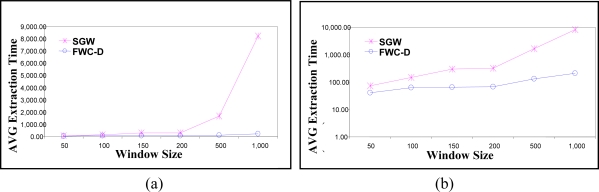
Execution time of the Integrity check algorithm at the receiver side. (**a**) Linear Scale, (**b**) Logarithmic Scale.

The execution time of the watermark extraction and integrity check is calculated and averaged over 30 groups. Figure 11 shows the average extraction and integrity check response time in the y-axis. The X-axis represents the window size. The group size *Z* is varied from 50 to 1,000 data readings. Figure 11(a) shows the average execution time on linear scale. To show the performance gain at small window sizes, Figure 11(b) shows the average execution time in logarithmic scale. The results show that the proposed watermarking technique is much faster than that of SGW. It also shows that the gain in the performance increases with increasing the window size. The figure shows that the FWC-D average extraction and integrity check response time at window size 1,000 is about 39 times faster than that required by SGW. Thus FWC-D significantly improves WSN response time.

**Figure 12.**
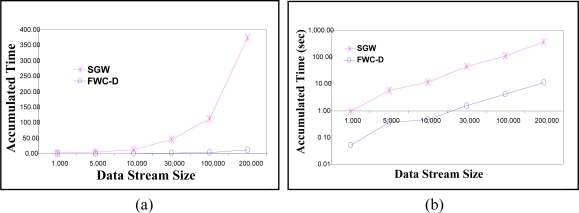
Accumulated execution time of the Integrity check algorithm at the receiver sensor. (**a**) Linear Scale (**b**) Logarithmic Scale.

The experiments in Figure 12(a) show the accumulated execution time of the watermarking extraction and integrity check algorithms. The Y-axis represents the accumulated processing time and the X-axis represents the number of data readings processed since the start of reception. Recall that we use the accumulated execution time as an indication of the expected power consumption at the sensor. The group size *Z* is set to 200. The FWC-D algorithm includes the following operations: constructing the data groups, generating and attaching the group delimiter *D* and serial number *SN* at the end of each window, watermark computation of group *g_i_*, extracting the embedded watermark from group *g_i−1_*, and comparing the extracted watermark against the reconstructed watermark. The results show that cost of the FWC-D is about 34 times less than the cost required by SGW.

## 7. Conclusions and Future Works

We have proposed FWC-D, a lightweight algorithm that uses a digital watermarking technique to provide data integrity for wireless sensor networks. FWC-D organizes data readings in groups with constant sizes and links group with each other by storing the watermark of the group in the following group. The watermark of group *g_i_* in FWC-D is formed using the hash function *HASH()*, which is applied to the concatenation of all data elements in group *g_i_* along with a secret key *K*, known only to the sender and receivers, and a group serial number *SN*. The watermark is stored in the least significant bits of the data readings in of group *g_i−1_*. FWC-D uses a group delimiter to keep the sender and receiver synchronized and avoids any ambiguity in case of data insertion or data deletion. The delimiter value *D* can take any value that does not occur in the data stream. In addition to the group delimiter *D*, FWC-D generates a serial number *SN* that will be attached and stored with each group and this will help the receiver determine how many group insertions or deletions occur in case of group insertion or deletion attacks.

We have provided a detailed security analysis for the proposed techniques and compared it with relevant prior works. We have also evaluated the cost, in terms of the execution time, of the proposed watermarking scheme and compared it with the execution time of the SGW one [12]. The experiments showed that the proposed schemes have much less computational overhead (one to two orders of magnitude less compared to the SGW scheme) and thus, can significantly improve the WSN lifetime. In the future, we plan to study semi fragile watermarking, which tolerates non-significant small changes, possibly caused by communication interference, but detect significant changes due to unauthorized alteration.

## Figures and Tables

**Table 1.** Notations and Parameters.

**Symbol**	**Description**

*HASH ()*	Secure hash function
*K*	Secret key
*g_i_*	The *i^th^* group of data stream
*S_i_*	A single reading value
*Z*	The number of data readings per group
*SN*	The serial number inserted in each group
*x*	The size, in bit, of the serial number
*W_i_*	The watermark of the *i^th^* group
*D*	Group delimiter
